# Microbial-Derived Biopolymers: A Pathway to Sustainable Civil Engineering

**DOI:** 10.3390/polym17020172

**Published:** 2025-01-12

**Authors:** Govindarajan Kannan, Evangelin Ramani Sujatha, Abdullah Almajed, Arif Ali Baig Moghal

**Affiliations:** 1 Department of Civil Engineering, Mepco Schlenk Engineering College, Sivakasi 626005, Tamil Nadu, India; rajkannan93@gmail.com; 2 Centre for Advanced Research in Environment, School of Civil Engineering, SASTRA Deemed University, Thanjavur 613401, Tamil Nadu, India; 3 Department of Civil Engineering, College of Engineering, King Saud University, Riyadh 11421, Saudi Arabia; alabduallah@ksu.edu.sa; 4 Department of Civil Engineering, National Institute of Technology, Warangal 506004, Telangana State, India

**Keywords:** microbial-derived biopolymer, ground improvement, building construction, water treatment

## Abstract

Modern innovations increasingly prioritize eco-friendliness, aiming to pave the way for a sustainable future. The field of civil engineering is no exception to this approach, and, in fact, it is associated with almost every sustainable development goal framed by the United Nations. Therefore, the sector has a pivotal role in achieving these goals. One such innovation is exploring the possibilities of using nature-friendly materials in different applications. Biopolymers are substances that are produced either by the chemical synthesis of natural materials or by the biosynthesizing activities of microorganisms. Microbial-derived biopolymers are known for their non-toxic and nature-friendly characteristics. However, their applications are mostly restricted to the field of biotechnology and not fully explored in civil engineering. This article reviews various microbial-derived biopolymers, focusing on the types available on the market, their source and properties, and more importantly, their wide range of applications in the civil engineering field. Additionally, the article explores the prospects for future research and the potential for the practical implementation of these techniques in the pursuit of a sustainable future.

## 1. Introduction

Civil engineering is a diverse field, and it is often misunderstood that the activities of this sector are restricted to just the design and construction of buildings. In reality, the sector encompasses a wide range of activities, including soil stabilization, pavement design and construction, water treatment, supply, and disposal, solid waste management, and the monitoring of air and water pollution. Although material manufacturing is not directly associated with civil engineering, the production of materials like cement, brick, lime, steel, etc., is often considered a related activity within the sector.

Material-based innovations typically offer two distinct solutions. First, they can serve as partial or complete replacements for conventional materials. For instance, in the case of cement, since its invention in 1824, it has been an indispensable material in the civil engineering sector. From infrastructure development to pavement construction, modern demands rely heavily on cement, a role previously fulfilled by lime. Yet, in recent times, more concerns have been raised regarding the usage of cement and lime in terms of their carbon emissions. It is reported that the production of one ton of cement and lime emits approximately 0.73–0.99 tons and 1.19–1.5 tons of CO_2_, respectively, which is a huge quantity considering the current market demands [1]. Although it is not possible to completely replace cement in building construction, geotechnical scientists started exploring the possibilities of using alternatives like biopolymers [2,3], microbe-induced calcite precipitation [4], geopolymers [5], etc., for soil stabilization. But there is another concern with conventional materials’ usage that has not yet been properly brought into the limelight. It is reported that increasing demand poses a serious threat to the faster depletion of natural raw materials (limestone) [6]. Hence, an alternative is always necessary, with green concrete appearing as a viable option [7]. The second advantage of material-based innovations lies in their ability to offer additional benefits beyond those provided by traditional materials. For instance, research has demonstrated that soil treated with biopolymers can adsorb heavy metals, a capability not achievable with cement treatment [8]. As stated earlier, it is not possible to completely eradicate cement and lime, as they have more benefits despite these drawbacks. Yet, as a step towards a sustainable future, researchers have started proposing alternative eco-friendly methods that would be essential for greater change in the distant future.

Several alternative techniques are under development for every application, but not all of them are nature-derived methods. For instance, several chemical flocculants are available on the market and may be introduced in the future also for water treatment. However, a recent study showed that a microalgal-based biopolymer can flocculate micro- and nanoplastics in wastewater treatment [9]. Hence, it is necessary to find the most suitable alternative that would be both effective and safe for the environment.

Biopolymers are substances that are produced either by the chemical synthesis of natural materials or by the biosynthesizing activities of microorganisms [10]. For instance, chitosan, a cationic polysaccharide, is formed by the chemical synthesis of exoskeletons of crustaceans like crabs and shrimp [11], whereas xanthan gum, an anionic polysaccharide, is produced by the fermentation of sugar by *Xanthomonas campestris* bacteria [12]. Although initial studies on biopolymer applications in civil engineering date back a few decades [13], active research on this topic started just a decade ago. Since then, researchers have found numerous applications of biopolymers for civil engineering, such as additives in concrete [14], additives for soil stabilization [15,16], etc. To date, many review articles have been published that summarize the results of biopolymer addition for soil stabilization or water treatment. For instance, Chang et al. (2020) conducted a comprehensive review of soil property modification with various biopolymer treatments [17]. They also discussed the potential applications of biopolymer soil stabilization and its environmental and economic impacts. Similarly, Elgarahy et al. (2023) critically analyzed various studies that utilized biopolymer composites for water treatment [18]. Several similar review articles have been published in the past that discuss the use of biopolymers for specific civil engineering applications. In most cases, the focus is on biopolymers in general, so not much attention has been given to specific categories.

Hence, the present article advocates for the application of microbial-derived biopolymers, a specific class of biopolymers, as an alternative to conventional methods in three different divisions of civil engineering, namely building construction, environmental engineering, and geotechnical engineering. Unlike traditional review articles which merely list all the available literature, this article compiles the crucial literature and focuses on the application point of view. However, material origin, their gelation properties, and their reaction mechanism are briefly discussed to aid in the understanding of these biopolymers’ role in the specified applications.

## 2. Microbial-Derived Biopolymers—Sources and Properties

As the name suggests, biopolymers are made of repeating monomer units derived from natural sources such as plants (e.g., guar gum), animals (e.g., chitosan), and microorganisms (e.g., xanthan gum). This study, however, focuses on the properties and applications of biopolymers that are influenced by microorganisms during one of their production stages. Based on the method of polymerization and charges, biopolymers can be classified as cationic (e.g., chitosan), anionic (e.g., xanthan gum), and non-ionic (e.g., guar gum). Based on the polymer backbone, they can also be classified as polysaccharides, peptides, and polynucleotides (e.g., deoxyribonucleic acid). Microbial-derived biopolymers can be sourced from bacteria (e.g., xanthan gum), fungi (chitin), or algae (Alginate). Table 1 lists some common microbial-derived biopolymers that have commercial applications in the industry.

Some of these microbial-derived biopolymers are synthesized based on a common fermentation process, with mild variations. For instance, the synthesis of xanthan gum starts with medium preparation, using carbon and nitrogen sources and other buffer chemicals. Then, the fermentation process is initiated with a suitable microorganism at optimum temperature and pH for a specific period. Once the fermentation process is over, the precipitated biopolymer is separated and purified by suitable techniques.

Most of the listed biopolymers are viscous, except for a few. Xanthan gum tends to form a viscous gel even in cold water [28], whereas gellan, in the presence of metallic ions, forms a gel when the temperature is greater than 40 °C [29]. On the other hand, pullulan, welan, and β-glucan (anionic derivative), though soluble in water, do not form any gel [8,20,30]. Curdlan is insoluble in water but soluble in an alkaline solution [31]. Alginate is water-soluble but can form a gel only in the presence of cross-linking agents [32]. Each of them has unique applications in various civil engineering practices, forming the basis of this review.

## 3. Applications in Building Construction

Most construction industry activities are centered in and around concrete. In this regard, some microbial-derived biopolymers have excellent applications in modifying the properties of concrete.

### 3.1. Viscosity Enhancer and Thickening Agent

Microbial biopolymers have a variety of applications in the construction sector. Viscosity enhancers can prevent separation in concrete mixes. In recent times, researchers have shown that microbial-derived biopolymers can act as effective viscosity enhancers. In this regard, xanthan gum is widely known for its viscosity-enhancing characteristics in concrete [33]. Masoumi et al. (2023) showed that the addition of just 0.2% xanthan gum to self-compacting concrete has many beneficial effects [34]. Xanthan gum can slow the hydration process and prevent the premature formation of gel materials. Further, xanthan gum reduces the porosity, leading to denser concrete and thereby improving its durability characteristics. Similarly, Zhang et al. (2016) showed that welan gum can act as a good thickening agent, with a negligible impact on the compressive strength [35]. Their microstructural investigation showed the use of welan gum for a threaded network that deforms with shear and unfolds again when shear is removed. Further, a recent study by Li et al. (2024) showed that both xanthan gum and welan gum influence the bleeding rate of concrete, which is greatly affected by temperature. However, the above authors stated that the presence of these gums would reduce the density of hardened concrete, which contradicts the findings of Masoumi et al. (2023). Hence, it should be understood that the effects of these gums are case-specific.

### 3.2. Accelerator

Accelerators are chemicals that, when added to a concrete mix, speed up the achievement of early strength and the setting process. This is much more useful when construction takes place in cold weather, where the hydration process is usually slow, to keep up with a fast construction schedule where early formwork removal is necessary. Engbert et al. (2020) showed that adding calcium alginate to aluminate cement accelerates the formation of calcium aluminate hydrate and improves the early strength. However, the authors suggested the addition of a superplasticizer to reduce the viscosity effect of calcium alginate [36].

### 3.3. Reinforcement Coating

Structures near the sea are easily affected by reinforcement corrosion. It is common practice to coat the reinforcement with a suitable material before concreting to reduce the corrosion effect. Roux et al. (2014) attempted to use a bacterial biopolymer produced by *Lactobacillus reuteri* as a corrosion inhibitor for concrete [37]. The authors reported that, irrespective of the additive dosage, the cathodic reaction was delayed due to the coating of the biopolymer over the reinforcement. However, the coating caused around 10% reduction in the compression and bending tests on the concrete after 90 days, due to improper gripping between the concrete and the reinforcement bars.

### 3.4. Crack Healing

Several chemical adhesives and sealants are available on the market to solve building cracking issues. However, these chemicals can be toxic at times, and several researchers are exploring how to overcome this issue naturally. One well-researched solution to this problem is the use of microbial-induced calcite precipitation [38], where bacteria produce calcium carbonate to heal the cracks. In a similar line of research, Zhang et al. (2023) showed that an alginate variety of biopolymers extracted from activated sludge exhibited around 86.5% crack closure in bricks with a mere 0.5% addition [39]. However, a marginal reduction in the flexural strength was noticed when compared with commercially available crack-healing agents. Recently, Valli and Kumar (2024) showed that a combination of cement and xanthan gum and tartaric acid has good crack-healing properties [40]. Additionally, the concrete performed well in terms of mechanical properties.

### 3.5. Biopolymer Bricks

Muguda et al. (2020) tested the effectiveness of using xanthan gum in earthen construction materials [41]. They showed that xanthan gum, due to its ionic bonding, enhanced the mechanical properties of the brick soil and also ensured favorable durability. Arab et al. (2021) prepared bricks using sand, sodium alginate, and the enzyme-induced carbonate precipitation (EICP) method [42]. The maximum dosage of sodium alginate was just 1.5% in this case. It was observed that the presence of sodium alginate enhanced the compression and flexural strength of the bio-bricks. Microscopic analysis indicated that the EICP, along with the sodium alginate, formed large calcite crystals in the inter-particle contacts, which greatly helped in enhancing the strength. Further, sodium alginate alone established a gel link with the soil particles which added an advantage to the mechanism. Recently, Kabalan et al. (2024) showed that the addition of 8% bacterial cellulose to bentonite enhanced the compression strength by 6.49 times, suggesting that, for bacterial cellulose stabilization, it is more suitable to use soil for earthen construction materials [43].

## 4. Applications in Environmental Engineering

### 4.1. Water Treatment

Usually, water undergoes a series of treatments before being suitable for drinking. Similarly, wastewater from any source undergoes a treatment process before being released in a major water source, such that the effluent should not affect the water body. Coagulation and flocculation are important stages of water treatment, where the particles or dust adhere together to form a bulky mass and settle down. Although several chemical coagulants and flocculants are available on the market, researchers have started introducing natural materials as alternatives to this process. Microbial-derived biopolymers are one such alternative to the synthetic chemicals used in this process.

Ghimici et al. (2010) showed that modified pullulan can be used as a flocculant in water treatment [44]. In a similar line of research, Chao et al. (2019) showed that a combination of aluminum chloride and carboxymethyl pullulan acted as an effective coagulant for kaolin [45]. Here, these materials complemented each other in the coagulation reaction, where aluminum chloride neutralized the charge and carboxymethyl pullulan acted as an adsorbent. Sudirgo et al. (2023) checked the dye removal efficiency in Congo red wastewater using xanthan gum as a coagulant-aid for poly aluminum chloride (PAC) [46]. The results showed that the removal efficiency of PAC increased from 81.16% to 93.81% by adding just 2 mg/L of xanthan gum. Also, it should be noted that, both in the case of Chao et al. (2019) and Sudirgo et al. (2023), the microbial-derived biopolymer could not offer an effective performance as a stand-alone additive, and aluminum chloride was essential in both cases for better results. Alginate as such cannot be used for water treatment; however, calcium alginate is very effective for water treatment. Saranya et al. (2022) showed that adding calcium chloride can help form calcium alginate to act as a coagulant for water treatment [47]. The calcium ions created charge neutralization, whereas the alginate facilitated coagulation. A turbidity removal of 93% was achieved with just 6 to 10 mg/L of alginate. Microplastics are one of the biggest threats to the environment, and, recently, many researchers have focused on effective methods to subside microplastic contamination. Rodrigues et al. (2024) showed that bacterial cellulose hydrogel from *Komagataeibacter saccharivorans* can effectively remove microfibers with 93.6% efficiency. Further, bacterial cellulose hydrogel can act as a flocculant with a minimum efficiency of 86.1%. These examples clearly show that microbial-derived biopolymers can be adopted as an effective additive in water treatment.

### 4.2. Heavy Metal Attenuation

Heavy metals are toxic substances that, even at a very low concentration, could be a dangerous threat to life. In general, heavy metals can be encountered in two scenarios. The first features heavy metal ions dissolved in water. The second concerns heavy metal that permeates into the ground from disposal sites, damaging both the soil and the ground. Numerous research studies were carried out in the past to prove that almost every biopolymer listed in Table 1 has the efficiency to adsorb heavy metals. Several review articles were published in the past that discuss the mechanism of heavy metal attenuation by biopolymers in both soil and water media [48,49]. Recent research largely involves the chemical modification of these biopolymers to have a better adsorption efficiency. However, the list in Table 2 shows limited examples of complete heavy metal adsorption using just the microbial-derived biopolymer without any other secondary additives.

## 5. Applications in Geotechnical Engineering

Out of the three categories in this review, geotechnical engineering is the most explored domain within biopolymer research. The reason is the ability of biopolymers to interact with clay and bind the soil particles. The geotechnical engineering properties of microbial-derived biopolymer-stabilized soil can be categorized into three major categories, namely strength improvement, permeability reduction, and compressibility characteristics. In addition to this, biopolymers have been used to subside soil erosion as well.

### 5.1. Strength Improvement

The strength of soil plays a crucial role in selecting the bearing medium for a structure. In many situations, the soil may not have enough capacity to bear the structure. In such cases, strength enhancement would be necessary. Studies on biopolymer-based ground improvement originated with the work of Yang et al. (1994). They used 3% xanthan gum solution- and 2% sodium alginate-treated soils, comparing the performance of bacterial soil stabilization, and reported that the cross-linking of soil–biopolymer matrices can enhance the strength [53]. Biopolymer soil stabilization depends on various influencing factors like soil type, biopolymer type, dosage, interaction mechanism, etc. Among all biopolymers, several strength enhancement studies were reported on xanthan gum-treated soil.

In a coarse-grained cohesionless soil, the viscous gel of xanthan gum dries in the soil matrix, and the firm binding contributes to the strength improvement of sand [54]. Lee et al. (2019) treated poorly graded sand with xanthan gum [54]. A significant increase in cohesion by 75.7 times and a negligible increase in the friction angle by 0.15 times were observed from the results of the triaxial test [54]. Cabalar and Canakci (2011) reported that a 5% xanthan gum solution offered a 2.88 times shear strength improvement in sand. However, their experiments showed insignificant strength variations with time and concluded that the curing period had a negligible effect on the strength of xanthan gum-treated sand [55]. Lee et al. (2021) studied the shear mobilization behavior of xanthan gum treatment in poorly graded sand [56]. The results showed that the biopolymer offered intergranular bonding, which weakened with excess moisture content. This shows that the moisture content of biopolymer-treated soil has a crucial role in strength improvement. β-glucan, welan, and pullulan, due to their inability to form viscous gel, are not effective in enhancing soil shear strength parameters. Fatehi et al. (2019) reported that an initial strength test on 1% sodium alginate-treated sand increased the UCS by 23.5%. A microstructural investigation showed that the polymer bound itself to the sand and consequent drying over time improved the strength [57]. Similarly, Chang and Cho (2016) tested the behavior of sand treated with gellan gum [19]. They observed that the gellan gum treatment was much more effective in the dry state than in the submerged state. The angle of internal friction increased by 70.7%, whereas the sand gained a cohesion of 166.2 kPa with 5% gellan gum addition. The strength improvement was attributed to the densification and aggregation effect created in the soil by the tensile gellan hydrogel. In comparison, the author stated that the strength of a 2% gellan gum treatment was equivalent to an 8% cement treatment after 7 days.

In the case of cohesive soil, clay, due to its surface charge, has a tendency to interact with biopolymers, which results in enhanced strength. Chang et al. (2015) showed that 1% xanthan gum improved the strength of clay by 4.8 times over 28 days [12]. Similarly, Singh and Das (2019) showed that 1% xanthan gum offered a 93% increase in the UCS of the highly plastic silt [58]. This increase in strength accounts for cation bridging between clay and xanthan monomers [58,59]. The result also infers that strength improvement greatly depends on soil type rather than just biopolymer dosage. Bozyigit et al. (2021) compared the effect of moisture content on xanthan gum-treated soil [60]. Their results revealed that the strength increased by 5.23 times with 2% xanthan gum over 90 days. An excess moisture content beyond 25% induced ductile behavior in the soil and reduced the strength.

**Figure 1:**
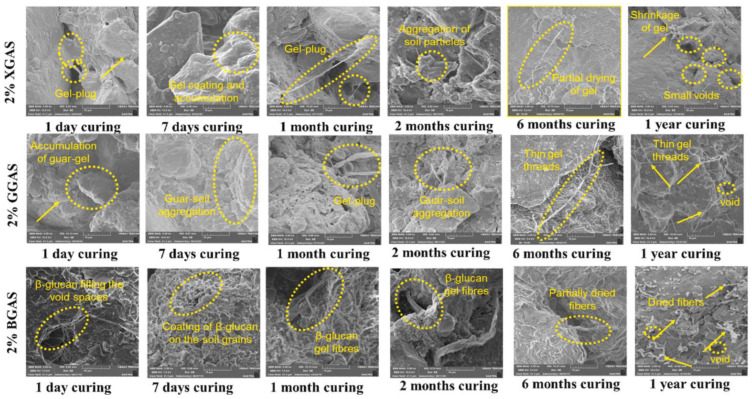
Mechanism of biopolymer interaction with soil (XGAS—xanthan gum-amended soil; GGAS—guar gum-amended soil; and BGAS—Bela glucan-amended soil). Reprinted with permission from ref. [8]. Copyright 2024 Elsevier.

Chang and Cho (2012) stabilized fine-grained soil with varying doses of β-glucan and observed a 3.10 times strength improvement over 28 days [61]. Kumar and Sujatha (2020) reported that 2% β-glucan could effectively increase cohesion in lean clay by 8.38 times [62]. The double layer of water formed an ionic bond with the anionic biopolymer and negatively charged clay minerals to increase the strength. The additive reduced the compression by 52.1% as the glucan fibers bonded to the soil matrix well. The comparison of the mechanism of xanthan gum, β-glucan, and guar gum interaction with the soil, as observed by Kumar and Sujatha (2020), is shown in Figure 1.

Bakhshizadeh et al. (2022) showed that sodium alginate increased the UCS of highly plastic clay by 90.13%. The interaction between soil and sodium alginate created a sodium aluminum silicate hydrate with the clay minerals forming hydration gels, resulting in strength improvement [63]. Similarly, Soltani et al. (2021) treated clay with sodium alginate and rubber crumbs [64]. The addition of 1% sodium alginate solution increased the UCS by 1.32 times over 7d. Similarly, the addition of 5% rubber shreds with 1% sodium alginate-treated soil showed a negligible 4.9% strength improvement. The sodium alginate increased the strength by the “flocculation-reinforcement” mechanism, which reduced the voids and increased the aggregated soil size to offer more strength.

Similarly, gellan gum also showed effective strength improvement in terms of shear strength parameters when used in pure clay. Chang and Cho (2019) showed that the addition of 4% gellan gum to pure clay improved the cohesive strength by 5.88 times and the friction angle by 0.64 times [65]. The tensile strength of gellan gel and the conglomeration effect of gellan gum with the clay increased the shear strength parameters. Further, the authors stated that the strength improvement would be even more effective in sand–clay mixtures.

### 5.2. Permeability Reduction

Permeability denotes the ease of water flow in the soil. Permeability is an important parameter that determines the choice of soil for an earthen dam core or a clay liner material. The interaction mechanism of soil with water during permeability is the same as that of the strength test under saturated conditions. Kumar and Sujatha (2021) monitored the permeability of a sand–clay mixture treated with xanthan gum and β-glucan for up to 1 year [8]. In all cases, the permeability was more than 1000-fold less than untreated soil with just 0.25% biopolymer treatment. The major mechanism here was the fact that the biopolymers created a bio-clogging mechanism that reduced the permeability. In xanthan gum, viscous gel coated the soil, thereby arresting the movement of water. However, it is interesting to note that β-glucan, although not viscous, developed fiber bundles that created cementitious bonding within the soil to reduce permeability. Chang et al. (2016) showed that even gellan gum showed a more than 1000-fold reduction in permeability with just 1% gellan gum addition to sand and silty sand. The authors noted that the further addition of gellan gum did not reduce the permeability beyond what had already been achieved and, hence, the initial dose could be labelled as the effective dosage for permeability reduction [66]. Bouazza et al. (2009) showed that a minimal addition of 0.5% sodium alginate in silty sand could reduce the permeability by 1000-fold in just 7 days. With aging, pore-clogging reduced the permeability by a further 10000-fold in 70 days [67]. Hence, it is clear that the viscous gels or fibrous threads of biopolymers play a crucial role in determining the permeability of soil.

### 5.3. Compressibility Characteristics

The compressibility of the soil plays a crucial role in determining the rate of settlement of soil in a structure. Kwon et al. (2023) studied the compressibility behavior of xanthan gum-treated kaolinite. The results showed that xanthan gum induced pore-clogging in the kaolinite, resulting in a delayed consolidation process [68]. Chang and Cho (2014) state that β-glucan effectively reduces the coefficient of consolidation, subsequently reducing the void ratio and, hence, the permeability [69]. However, the compression index in their study did not show much change. The authors reported that this modification was due to the clogging effect created by β-glucan in the soil. Hence, in general, it can be inferred that biopolymers offer resistance to compression due to their interaction mechanism with soil. Further, consolidation characteristics also determine the permeability of soil under pressure. Considering the advantageous effect of biopolymers, the permeability of treated soil is lower than that of untreated soil under similar loading conditions.

### 5.4. Erosion Resistance

**Figure 2:**
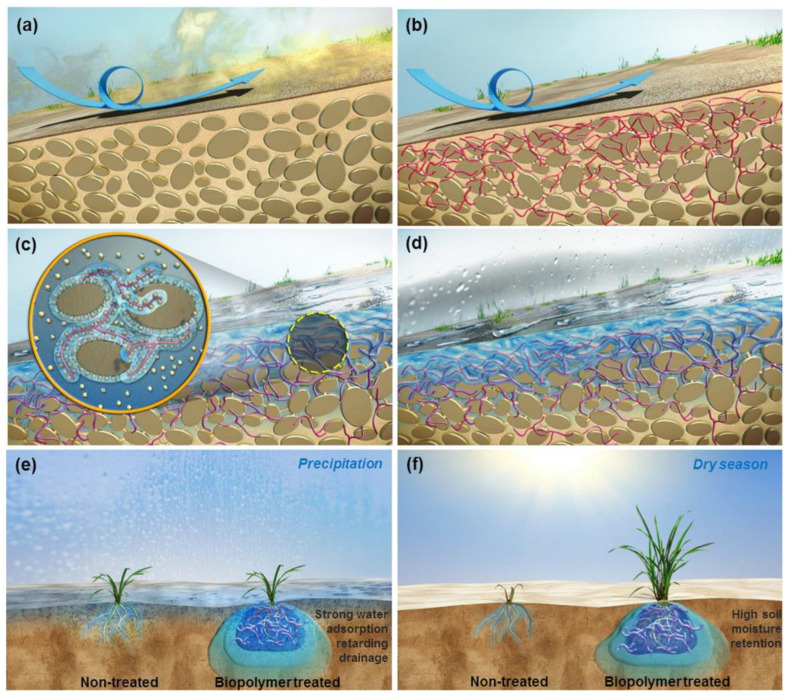
(**a**) Erosion of untreated soil, (**b**) erosion resistance offered by biopolymer-treated soil, (**c**) water-zone created for effective plant growth due to hydrophilic adsorption, (**d**) biopolymer coating the soil surface, (**e**) enhanced water adsorption during precipitation, and (**f**) moisture retention during the dry season. Reprinted with permission from ref. [72]. Copyright 2024 Elsevier.

Erosion refers to removing soil mass from a region due to agents like air and water. Continuous soil loss may alter the ecosystem completely, affecting the region’s fertility, cultivation pattern, etc. Recently, the ability of biopolymers to bind soil particles together found applications in wind erosion resistance as well. Ayeldeen et al. (2016) tested the effect of various biopolymer treatments in sand subjected to wind erosion [70]. The authors tested 500 g of sand exposed to a wind velocity of 150 kmph for 10 min in a chamber. The results revealed that a concentration of 0.25% xanthan gum as surface spraying resulted in only 1.67% loss under wet conditions and 5.93% under dry conditions, respectively, rather than the 3.33% and 10.73% losses recorded before treatment. The low-viscous xanthan gum gel covered the soil particles and prevented erosion, and the effect was reduced with an increase in the biopolymer’s dosage. Lemboye et al. (2021) conducted a similar test on sand sprayed with sodium alginate and tested at a velocity of 58.32 kmph for 33 min. The results revealed that there was no visible loss of sand after the exposure, and the authors noticed that any concentration between 0.5% and 3.0% created a crust of sand upon spraying, which prevented erosion [71]. Similarly, Chang et al. (2015) tested the effect of water erosion on 0.5% xanthan gum and β-glucan sand exposed to 8000 mm/hr rainfall intensity for 5 s [72]. Even here, the soil showed less erodibility at a lower biopolymer concentration of 0.5%. The authors claimed that the biopolymer coated the surface strongly, which, upon drying, created a solid mass which resisted erosion. Further, the authors showed that the addition of these biopolymers helped vegetation as well, meaning that it could serve as an effective tool against any type of erosion. The mechanism of erosion resistance in microbial-derived biopolymer-treated soil in the case of wind and water erosion is depicted in Figure 2.

## 6. Discussion on Suitability

Based on the articles discussed, it can be observed that not all microbial-derived biopolymers are used for every purpose. Several factors like the ability of the biopolymer to form a gel, its dosage, viscosity, etc., have a crucial role to play in every application. Also, it can be seen that, compared to environmental and geotechnical applications, microbial-derived biopolymers are not often used in construction industries. Further, the environmental applications of such microbial biopolymers are a vast topic to discuss, as the works which involved a combination of biopolymers and other materials were not under the scope of this research. From the geotechnical engineering point of view, almost all the biopolymers in the discussed studies were tested for their strength and permeability enhancement, whereas very few studies were carried out on the soil erosion front. Another drawback in geotechnical engineering applications compared to the other two is the rate of degradation of biopolymers. In building construction, the chances of concrete being exposed to a degradation medium are lower, and, even if exposed, there are no noticeable reactions. Similarly, if used for water treatment, these biopolymers are short-lived and, once their application for coagulation and adsorption is over, a new batch can be introduced. However, in geotechnical engineering, except for the case of wind erosion, this treatment is used for any stabilization application that needs to be long-lasting, which, however, is not possible, especially when the exposure medium is soil. This is a major reason why this procedure has not been introduced in practical applications yet.

## 7. Durability

The durability of a biopolymer soil treatment can be measured in two aspects: one is the strength reduction due to physical modification based on wet–dry and freeze–thaw cycle effects, and the other is the strength due to the biodegradation effect, during which the biopolymer itself is degraded. The literature suggests that biopolymer-treated soil undergoes strength reduction due to wet–dry and freeze–thaw cycles. For instance, Lee et al. (2022) stated that cohesionless soil is more susceptible to strength reduction even with 2% xanthan gum–starch treatment [73]. However, the presence of 15–25% fine content in the soil should maintain the strength within the safe limits for levee construction even after six wet–dry and freeze–thaw cycles. In the case of biodegradation, durability depends on the type of soil and the microorganisms present in it. Not many studies have been explicitly conducted to highlight this drawback; however, in a different study, Muchová et al. (2009) showed that bacteria in activated sludge can degrade xanthan gum and gellan gum in seven days [74]. However, this is a case of continuous degradation under ideal conditions. Similarly, Stachowiak et al. (2023) showed that sodium alginate–gellan gum beads biodegrade in 28 days when exposed to soil and seawater [75]. The authors mentioned that degradation is faster in the soil, as a wider range of microorganisms are present in it compared to seawater. In the case of soil stabilization, even though laboratory testing conditions generally show continuous strength improvement [76], field conditions may alter the degradation rate. In recent times, studies have claimed that the cross-linking of biopolymers can reduce the biodegradation rate [77]. However, these claims are limited to tissue and biomedical engineering applications, and not a lot of evidence is available to evaluate this for bulk civil engineering applications.

## 8. Environmental and Economic Concerns

**Figure 3:**
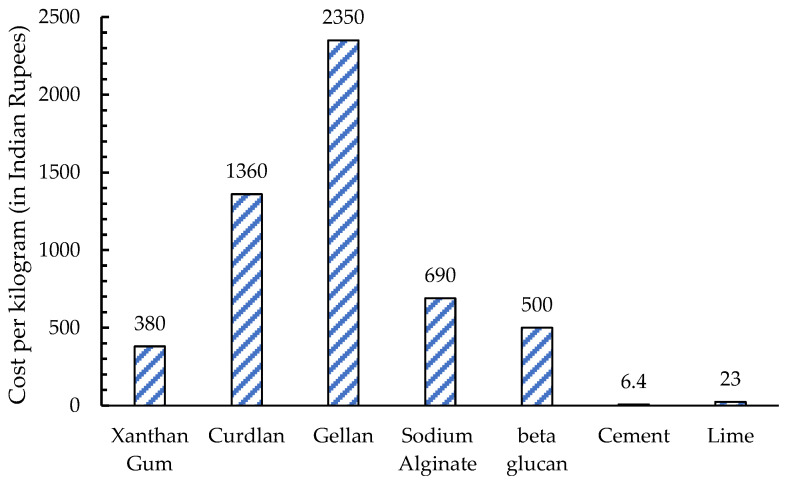
Cost comparison of various microbial-derived biopolymers with conventional soil stabilizers [79].

Although biopolymers can be produced by less-emissive techniques, commercial production at present has higher emissions for some biopolymers than cement. For instance, the average rate of carbon expected to be released from cement production is 0.7 ton CO_2_ e/ton [78], while the CO_2_ emitted with xanthan gum production is 1.67 ton CO_2_ e/ton [79]. In general, biopolymer techniques are less emissive because the amount of carbon emitted to produce the necessary quantity of conventional stabilizers (like cement) is higher than that required for biopolymer stabilization, as biopolymers require a smaller dosage. Hence, active research should focus on finding greener methods of producing biopolymers. Another important issue to be addressed is cost. Despite the advantages listed, the cost of biopolymers is still much higher than that of cement. Figure 3 shows the cost comparison of commercially available microbial-derived biopolymers and conventional stabilizers like cement and lime. It is evident from the data that even the cheapest biopolymer, xanthan gum, is nearly 59 times more expensive than cement and almost 15 times more expensive than lime. Although the dosage of microbial-derived biopolymers (or any biopolymer in this case) required for soil treatment would be lower (1% to 2%) compared to cement and lime (5% to 10%), the stabilization cost would still be high.

It should be noted that biopolymers, in general, are available at various grades, like cement, but no standard market price has been established for them. Hence, the price of these materials varies depending on the sellers and the production methods. The main reason for this scenario is that the commercial production of biopolymers is not as widespread as that of cement. However, given their promising applications in the civil engineering sector, where material demand is in bulk quantities, there is potential for a reduction in material costs in the future.

## 9. Conclusions

This review article summarized all the civil engineering applications of microbial-derived biopolymers in the building construction, environmental engineering, and geotechnical engineering domains. It was observed that xanthan gum is the most extensively researched microbial-derived biopolymer, followed by sodium alginate, particularly for applications in soil stabilization. Both of these biopolymers offer enhanced strength, reduced permeability, and resistance to wind erosion. Additionally, they are effective in attenuating heavy metals such as Pb, Cd, and Cu. Both alginate and welan gum contribute positively to the properties of concrete. In terms of water treatment, biopolymers have shown effectiveness as secondary additives in conjunction with primary chemical compounds. However, not all biopolymers have been thoroughly tested for every application. One significant reason for this is their cost; although microbial-derived biopolymers serve as green alternatives to conventional additives like lime and cement, they can be more than 50 times more expensive than cement. Another major concern regarding the application of biopolymers is their durability. The biodegradation of microbial-derived biopolymers is accelerated when exposed to organic soil or contaminated water. While research aimed at altering biodegradation rates is emerging, it remains largely confined to biotechnological applications. Therefore, it is recommended that future research focuses on developing cost-effective and less-emissive methods for producing these microbial-derived biopolymers, as well as on enhancing their durability for long-term use. Furthermore, detailed explorations of the potential of cross-linking these microbial-derived biopolymers with other additives, such as nanomaterials and other biopolymers, are essential to fully utilize their promise for commercial applications.

## Figures and Tables

**Table 1:** Source and properties of common microbial-derived biopolymers.

Biopolymer	Category	Ionic Nature	Source	Microbe Name	Ref.
Xanthan	Polysaccharide	Anionic	Bacteria	*Xanthomonas campestris*	[8]
Gellan	Polysaccharide	Anionic	Bacteria	*Sphingomonas elodea*	[19]
Welan	Polysaccharide	Anionic	Bacteria	*Sphingomonas* sp.	[20]
Pullulan	Polysaccharide	Non-ionic	Fungi	*Aureobasidium pullulans*	[21]
Curdlan	Polysaccharide	Non-ionic ^1^	Bacteria	*Alcaligenes faecalis*	[22]
β-glucan	Polysaccharide	Non-ionic ^1^	Bacteria	*Bacillus subtilis*	[23]
β-glucan	Polysaccharide	Non-ionic ^1^	Fungi	*Saccharomyces cerevisiae*	[24]
Alginate	Polysaccharide	Anionic	Algae	*Macrocystis pyrifera*	[25]
Hyaluronic acid	Polysaccharide	Anionic	Bacteria	*Streptococcus zooepidemicus*	[26]
Bacterial cellulose	Polysaccharide	Anionic^1^	Bacteria	*Cluconacetobacter xylinus*	[27]

^1^ Can exhibit a different ionic nature depending on the preparation methods.

**Table 2:** Heavy metal attenuation by microbial-derived biopolymers in soil and water.

Microbial-Derived Biopolymer	Medium	Dosage	Heavy Metals	Removal Efficiency	Remarks	Ref.
Xanthan gum	Soil	2%	Cd	Almost complete removal	-	[8]
			Zn		-	
			Cu		-	
			Pb		-	
β-glucan	Soil	2%	Cd	44.86%	-	[8]
			Zn	7.99%	-	
			Cu	11.42%	-	
			Pb	26.19%	-	
Gelan gum	Soil	2%	Ni	60%	-	[50]
Bacterial cellulose	Liquid	10 mg/mL	Pb	~80%	-	[51]
			Ni	~30%	-	
			Cd	~40%	-	
Sodium alginate (SA)	Liquid	1 g/L	Pb	~65%	5:1 (SA: heavy metal)	[52]
			Cu	~85%		
			Cd	~80%		

## Data Availability

No new data were created in this study. Data sharing is not applicable to this article.
